# Mapping Snakebite Epidemiology in Nicaragua – Pitfalls and Possible Solutions

**DOI:** 10.1371/journal.pntd.0000896

**Published:** 2010-11-23

**Authors:** Erik Hansson, Steven Cuadra, Anna Oudin, Kim de Jong, Emilie Stroh, Kjell Torén, Maria Albin

**Affiliations:** 1 Division of Occupational and Environmental Medicine, Lund University, Lund, Sweden; 2 Department of Occupational and Environmental Medicine, Skåne University Hospital, Lund, Sweden; 3 Departamento de Medicina Preventiva, Facultad de Ciencias Médicas, Universidad Nacional Autónoma de Nicaragua, Managua, Nicaragua; 4 Division of Occupational and Environmental Medicine, Umeå University, Umeå, Sweden; 5 Department of Occupational Medicine, Sahlgrenska University Hospital, Göteborg, Sweden; Liverpool School of Tropical Medicine, United Kingdom

## Abstract

**Background:**

Snakebites are a public health problem in Nicaragua: it is a tropical developing country, venomous snakes are present and there are reports of snakebites treated both in the formal and informal health care system. We aimed to produce an incidence map using data reported by the health care system that would be used to allocate resources. However, this map may suffer from case detection bias and decisions based on this map will neglect snakebite victims who do not receive healthcare. To avoid this error, we try to identify where underreporting is likely based on available information.

**Method and Findings:**

The Nicaraguan municipalities are categorized by precipitation, altitude and geographical location into regions of assumed homogenous snake prevalence. Socio-economic and healthcare variables hypothesized to be related to underreporting of snakebites are aggregated into an index. The environmental region variable, the underreporting index and three demographic variables (rurality, sex and age distribution) are entered in a Poisson regression model of municipality-level snakebite incidence. In this model, the underreporting index is non-linearly associated with snakebite incidence, a finding we attribute to underreporting in the most deprived municipalities. The municipalities with the worst scoring on the underreporting index and those with combined low reported incidence and large rural population are identified as likely underreporting. 3,286 snakebite cases were reported in 2005–2009, corresponding to a 5-year incidence of 56 bites per 100,000 inhabitants (municipality range: 0–600 cases per 100,000 inhabitants).

**Conclusions:**

Using publicly available data, we identified areas likely to be underreporting snakebites and highlighted these areas instead of leaving them “white” on the incidence map. The effects of the case detection bias on the distribution of resources against snakebites could decrease. Although not yet verified empirically, our study provides an example of how snake bite epidemiology may be investigated in similar settings worldwide at a low cost.

## Author Summary

Snakebites have recently been recognized as a neglected cause of human suffering and death worldwide. Many bites are treated by traditional practitioners and thereby not recorded by the health care system. This leads to a lack of reliable epidemiological data and is identified as a major obstacle in dealing with this health problem. Household surveys are recommended for finding the true snakebite incidence, but the countries where snakebites are frequent are usually poor, meaning that this method is often too expensive. The usage of data reported by the health care system could then provide a necessary option when locating and estimating the snakebite problem. However, this data could be biased and lead to implementation of unfair policies. In this study, we use publicly available data about environmental, socioeconomic and health-care related variables and incidence reported from health care facilities to create a map of where underreporting could be suspected, either because of the presence of factors favouring underreporting or by a comparatively low reported incidence. By high-lighting these areas, the reported statistics are put into a context and the decision-maker is able to make a less biased decision on where to locate research, preventive and therapeutic resources.

## Introduction

Snakebite accidents are on the WHO list of neglected tropical diseases since April 2009 [1], [2]. This neglect is understandable since most snakebite victims are rural residents in tropical countries [3] who lack financial resources and political power. Furthermore, many bites are treated by traditional practitioners [4]–[6], meaning that information about some cases does not become available to the health authorities, hindering effective and fair distribution of health care resources such` as antivenom [7]. A study of underreporting of fatal snakebites in hospital data compared to death registers in Sri Lanka [8] found that there were three times as many snakebite deaths than registered fatal snakebites in hospital statistics.

In Nicaragua, a tropical developing country with 44% rural population [9], the most dangerous snake species is *Bothrops asper*, a pit viper restricted to the wet, lowland east part of the country [10]–[12]. Research from neighbouring Costa Rica claims that *Bothrops asper* is well adapted to environments affected by small-scale agriculture, making snake-human encounters frequent during agricultural activities in the fields and close to rural dwellings [13]. Given that Costa Rica is a more urbanized [14] and developed [15] country than Nicaragua, it could be assumed that the small-scale agriculture is an important factor favouring snake-human interactions also in Nicaragua. In the drier and more densely populated west part of the country, *Crotalus simus* is considered the most dangerous snake species ([16] and JM Gutiérrez, Instituto Clodomiro Picado, University of Costa Rica, December 2009, personal communication). Many snakebite victims in Nicaragua [17] and the neighbouring countries [18] are adult men, likely because they are more likely to work in the agricultural setting where they are exposed to snakes. There are indications that snake envenoming could be a neglected health problem in Nicaragua as there are reports of 1) areas with high snakebite incidence [17], 2) traditional treatment of snakebites [19], [20], 3) problems with antivenin supply in some areas [21], 4) unequal distribution of health care resources [22] and 5) underreporting of other diseases in the country (e.g. pesticide poisoning) [23].

Household surveys are recommended when investigating snakebite epidemiology since data gathered by the health care system likely underestimates the real incidence [4]. Since household surveys are expensive, we tried to interpret the health care system data by producing a map of the reported incidence. However, simply mapping this incidence data could produce a map highly biased by case detection if there are geographical differences in healthcare accessibility and usage. This issue is important to address as it otherwise could lead to implementation of unfair and inefficient prioritizations that further neglect the disease of those that do not have access to healthcare. The reader of the hospital data incidence map must be able to evaluate the mapped data critically and assess where it is more likely to suffer from underreporting.

Our aim is to use publicly available data to identify municipalities where snakebites are underreported using two theoretical assumptions of how underreporting could be identified: (a) presence of factors facilitating underreporting (e.g. long distances to healthcare) and (b) lower reported incidence compared to what could be expected (i.e. low incidence compared to near-by and environmentally similar municipalities despite presence of risk group (rural residents)).

We also aim to describe the relationship between reported incidence, environmental and demographic factors and factors related to underreporting on municipality level as well as seasonal factors. Our hypothesis is that reported snakebite incidence varies with environmental and seasonal factors and is positively associated with rural population percentage and poor socioeconomic conditions up to a peak level where underreporting will be so extensive that reported incidence will start declining.

## Materials and Methods

### Material

**Figure 1:**
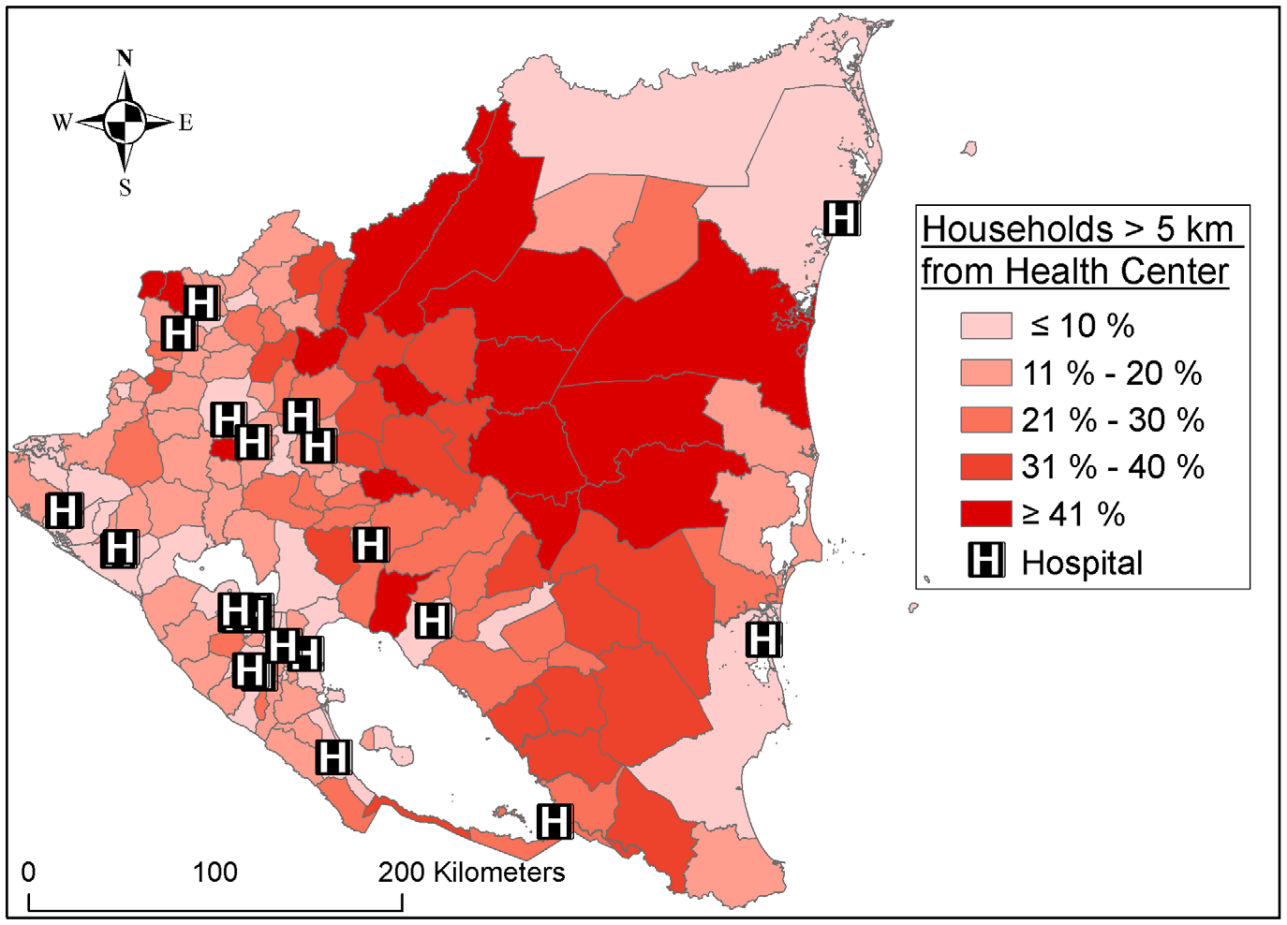
Distribution of hospitals and health centres in Nicaragua.

Data about environmental, demographic, socioeconomic and health care related factors were gathered from international and Nicaraguan organizations (Table S1) either as tables or maps. ArcGIS 9.3 (ESRI, 1999–2008) was used to extract and process map data. Information on the number of reported snakebite cases (non-fatal and fatal) each week during 2005–2009 from each municipality/health centre/hospital was obtained in December 2009 from the surveillance system Sistema Nicaragüense de Vigilancia Epidemiológica Nacional (SISNIVEN) (administrated by the Nicaraguan Ministry of Health, MINSA). Cases reported from health centres/hospitals, were counted as from the municipality where the health centre/hospital was situated. All but one municipality have one or more health centres. The hospitals are unevenly distributed (Figure 1).

### Categorization into regions with assumed similar snake prevalence

**Figure 2:**
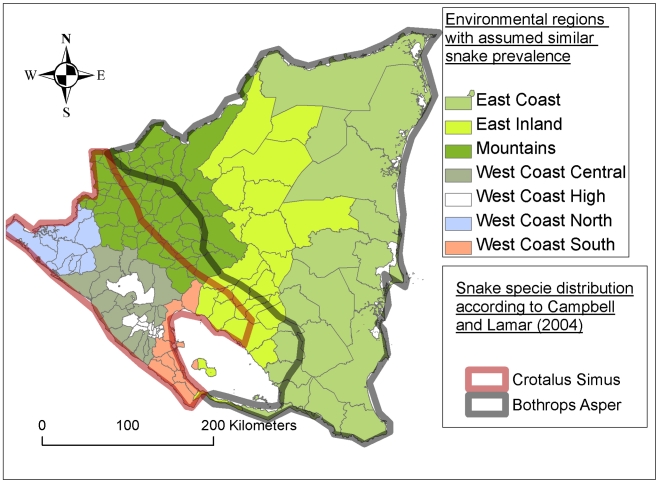
Environmental regions and snake distribution. Within the environmental regions, homogenous snake prevalence is assumed based on similarities in altitude, precipitation and geographical location (see Methods section).

In an iterative and visual process, ArcGIS and PASW Statistics 18 (SPSS Inc., 2009) were used concomitantly to create groups of municipalities within which similar snake prevalence was assumed based on homogeneity in altitude, precipitation and geographical location (Figure 2 and Table S2). With this method snake prevalence is considered the result of an interaction between altitude, precipitation and other factors for which geographical proximity is a proxy. Altitude, precipitation and vegetation type are parameters used when describing the distribution of snake species in Nicaragua in literature [12]. From now on these groups of municipalities will be referred to as “regions”.

### Identification of underreporting

The municipalities likely to be underreporting were identified by two approaches:

- **Identifying the presence of factors favoring underreporting through construction of an underreporting index:** Six municipality-level socioeconomic and health-care-related variables that were hypothesized to be related to snakebite underreporting were used: 1) distance to hospital from center of municipality (m), 2) households more than 5 km from health center (%), 3) population in poverty (%), 4) illiteracy (%), 5) area with more than 1 km to road (%) and 6) number of latest birth outside of health care system per inhabitant (Table S1 and S3). The municipalities were ranked according to each of the variables. The ranks of all of the variables were summed to create a rank index consisting of an aggregation of all the six variables with a possible maximum sum (best ranking for every variable) of 912 and a possible minimum sum of 6. This was done to let the different variables together give a more nuanced picture of the deprivation and remoteness of the municipalities than using only one of them. Because of lack of background knowledge or theoretical reasons to assume differently, all variables were given equal weight. We will refer to this index as the “underreporting index”.
- **Identifying a lower incidence than expected:** Within each region the municipalities were ranked depending on their reported incidence and percentage rural population. The municipalities that had a combination of being in the top 40% of the rural population percentage rank and in the bottom 40% of the incidence rank were identified as potentially underreporting. From now on these municipalities will be called “low-reporters”

### Statistics

The data was analysed with Poisson regression, chosen as it is suitable for group-level rates of events in a population. Egret for Windows 2.0 (CYTEL Software Corporation, 1999) was used for the analysis. The outcome variable was the total number of reported snakebites between 2005 and 2009 in each municipality. Municipality population size 2007 (projection from the 2005 census) was used as rate multiplier variable. As environmental variable, the regions constructed as described above was entered, i.e. a categorical variable describing the interaction between altitude, precipitation and geographical location. The Mountain region was used as reference category as it had the highest number of municipalities (n = 56). The percentage 1) rural population, 2) male/female ratio and 3) population <15 years were entered as continuous variables, as these were expected to be linearly associated with incidence. The underreporting index (described above) was entered as a categorical variable, as it was expected to be non-linearly associated with reported snakebite incidence. The index is associated with high rural population and thereby many bites, but also to factors hypothesized to favour underreporting that could be assumed to counteract this positive association and therefore make the association with incidence peak at a certain level. The most extreme categories of the underreporting index variable were allowed to consist of fewer municipalities in order to be able to discern effects in the ends of the distribution (Table S4). The second best category (good) was used as a reference category as this had the highest number of municipalities (n = 45). Incidence ratios (IR) with 95% confidence intervals were estimated from the model.

## Results

### Descriptive data

**Figure 3:**
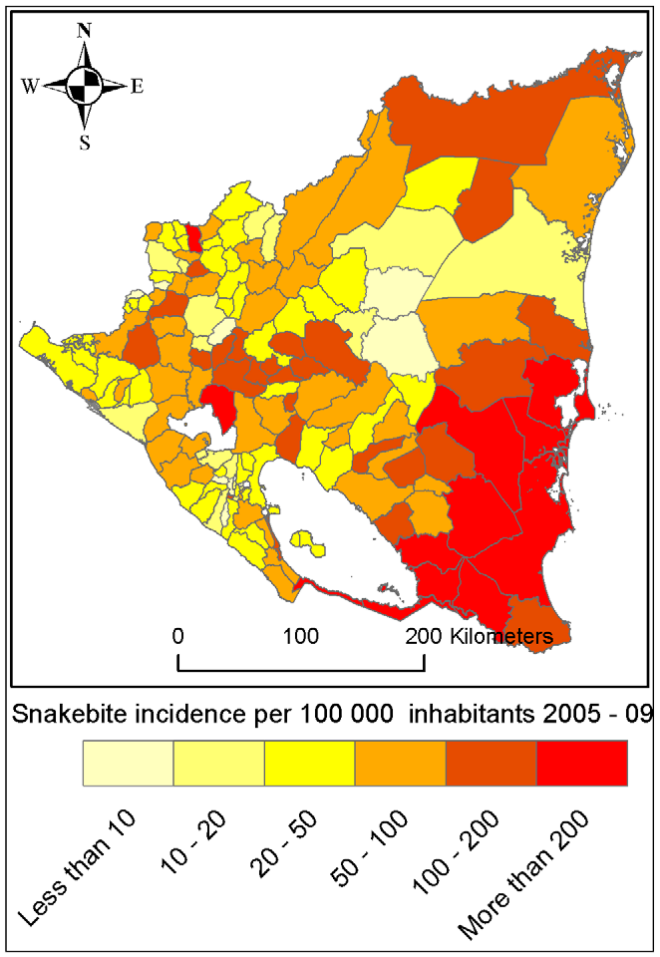
Snakebite incidence 2005–2009 according to data reported from the Nicaraguan Ministry of Health (MINSA).

**Figure 4:**
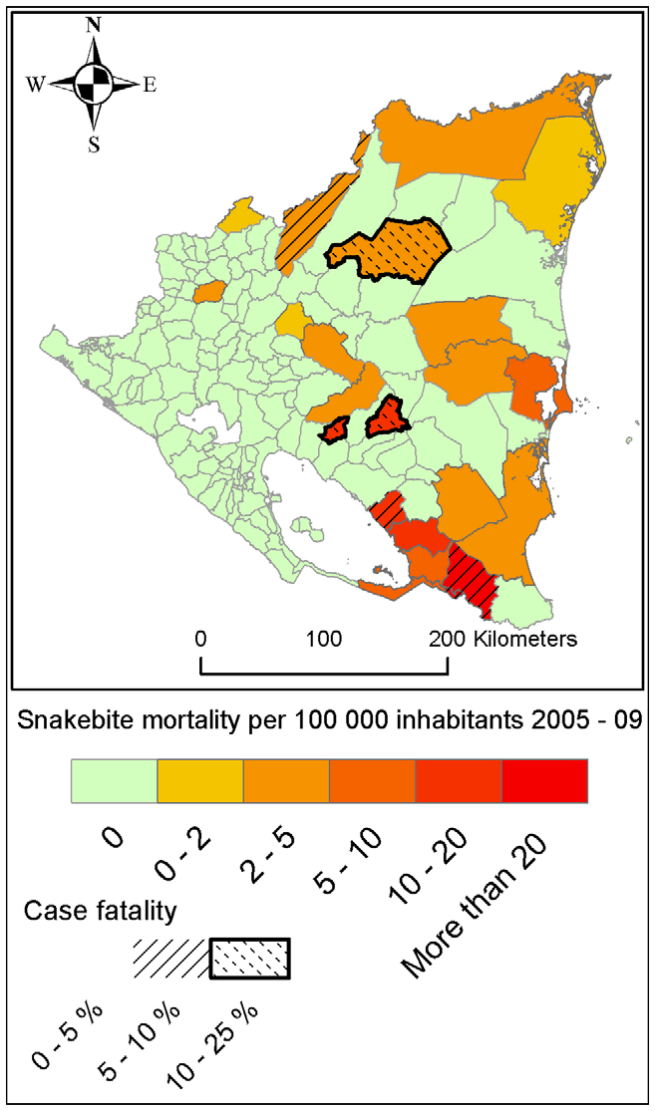
Snakebite mortality 2005–2009 according to data reported from the Nicaraguan Ministry of Health (MINSA).

**Figure 5:**
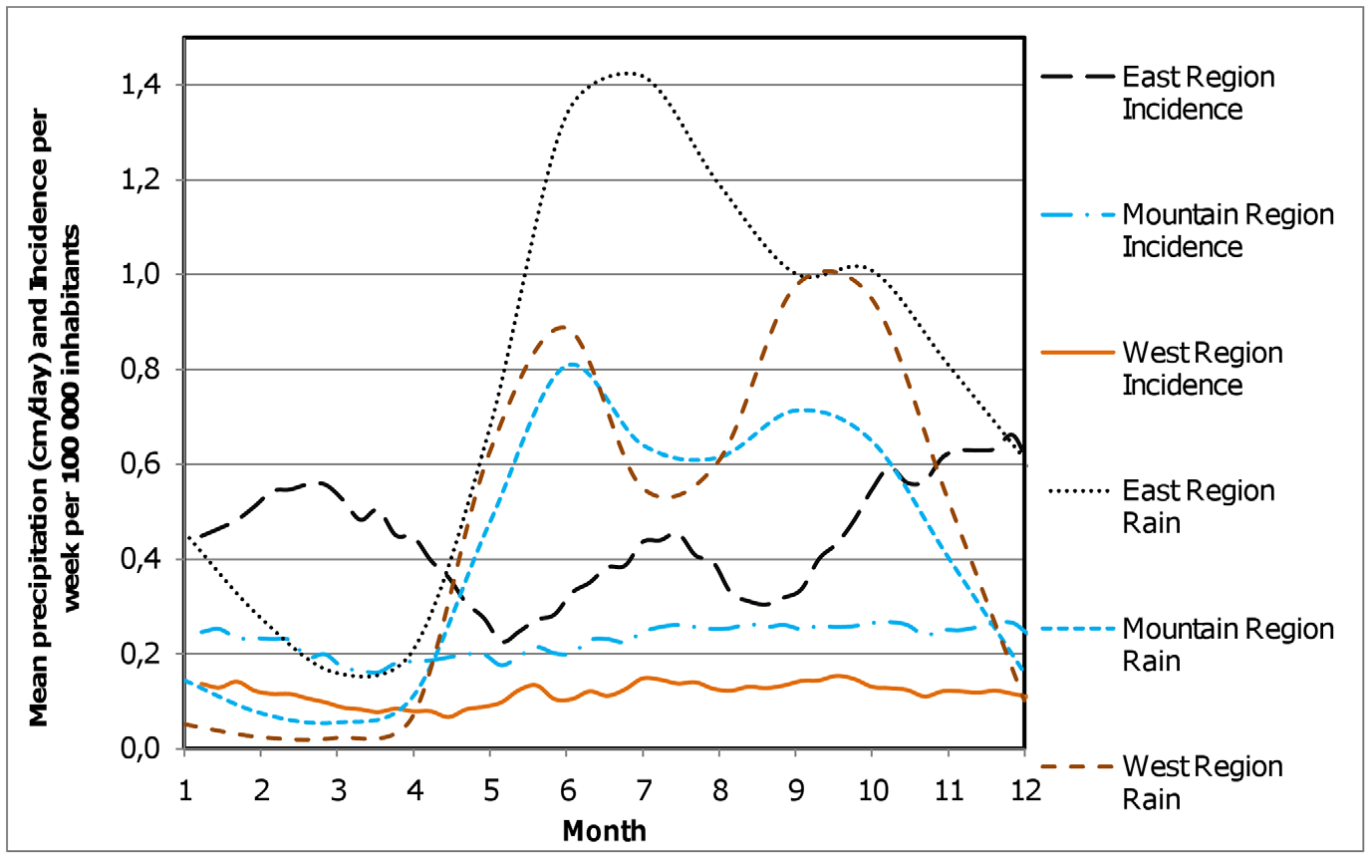
Seasonal distribution of rain and snakebites in three regions in Nicaragua. The regions are aggregations of the regions in Figure 2. East Region  =  East Inland and Coast, West Region  =  West Coast North, Central, High and South. In order to produce a smooth graph line, the seasonal variation is shown as a sliding one-week mean of the week, the two previous and the two following weeks (a total of five weeks).

Among Nicaragua's 5.9 million inhabitants (2007 population projection) [9], there were 3,286 reported snakebite cases between 2005 and 2009. This 5-year incidence of 56 snakebites per 100,000 inhabitants is unevenly geographically distributed; the highest incidence is seen in the south-eastern part of the country (Table 1 and Figure 3). In total there were 34 reported fatal snakebites in Nicaragua in 2005–2009, corresponding to 0.6 fatal cases per 100,000 inhabitants in 5 years and a 1% case fatality rate. All fatal snakebites were in the central or eastern part of the country (Figure 4). Snakebite incidence has a seasonal variation which is most pronounced in the east part of the country where the incidence almost triples between the lowest (May) and highest (December) months (Figure 5).

**Table 1:** 5-year snakebite incidence in environmental regions.

Environmental region	5-year incidence per 100 000 inhabitants
West Coast South	58.8
West Coast Central	24.1
West Coast North	39.8
West Coast High	26.8
Mountains	59.6
East Inland	46.8
East Coast	187.9

### Snakebite incidence ratio estimates in Poisson regression model

In the Poisson regression model, reported snakebite incidence ratios were strongly associated with environmental region (in the fully adjusted model ranging from 0.66 – 2.57) (Table 2). The highest incidence ratio was seen in the East Coast Region and the lowest in the West Coast High Region. In the fully adjusted model, a one percentage unit increase in proportion rural population increases the snakebite incidence by 0.1% (95% C.I. -0.2–0.4%). In a model without the underreporting index, a one percentage unit increase in rural population increases reported incidence by 1.0% (95% C.I. 0.7 – 1.4%). Male surplus (percentage more men) has a positive association with reported incidence (I.R. 1.02 95% C.I. 1.01 – 1.03). Percentage population <15 years of age is negatively associated with reported incidence in the adjusted model (fully adjusted I.R. 0.97 95% C.I. 0.95–0.98), but not in the univariate model where there is instead a positive association (I.R. 1.07 95% C.I. 1.07–1.08). The underreporting index is positively associated with incidence until a peak in the second last category consisting of the 11^th^–30^th^ worst municipalities (incidence ratio 1.72 95% C.I. 1.45–2.01), then it drops drastically in the category consisting of the worst ten municipalities (incidence ratio 0.97 95% C.I. 0.74–1.26) (Table 2).

**Table 2:** Snakebite incidence ratios of environmental regions, demographic variables and underreporting index categories in Poisson regression models.

		Terms entered separately			Adjusted for environmental region and demographic variables				Adjusted for all terms			
Term	n^1^	IR^2^	95% C.I.		IR^2^		95% C.I.		IR^2^		95% C.I.	
**Environmental region**												
West Coast South	11	0.99	0.84	1.15	0.96		0.81	1.13	1.30		1.10	1.55
West Coast Central	19	0.41	0.36	0.45	0.59		0.52	0.68	0.80		0.70	0.92
West Coast North	12	0.67	0.57	0.79	0.77		0.66	0.91	0.86		0.72	1.01
West Coast High	14	0.44	0.34	0.57	0.45		0.34	0.58	0.66		0.51	0.85
Mountains	56	Reference category				Reference category				Reference category		
East Inland	22	0.79	0.69	0.90	0.84		0.73	0.97	0.79		0.69	0.93
East Coast	18	3.16	2.90	3.44	3.68		3.30	4.11	2.57		2.29	2.87
**Demographic variables**												
Rural inhabitants (%)	-	1.017	1.016	1.018	1.010		1.007	1.014	1.001		0.998	1.004
Population <15 years (%)	-	1.07	1.07	1.08	0.96		0.94	0.97	0.97		0.95	0.98
Male surplus (%)	-	1.09	1.09	1.10	1.04		1.03	1.05	1.02		1.01	1.03
**Underreporting index category**												
Best	32	0.33	0.30	0.37	not included				0.38		0.33	0.44
Good	45	Reference category			not included				Reference category			
Medium	45	2.07	1.90	2.27	not included				1.71		1.53	1.91
Bad	20	2.14	1.92	2.39	not included				1.72		1.45	2.01
Worst	10	0.72	0.61	0.85	not included				0.97		0.74	1.26

1 Number of municipalities in category.

2 Incidence ratio.

### Detection of underreporting

**Figure 6:**
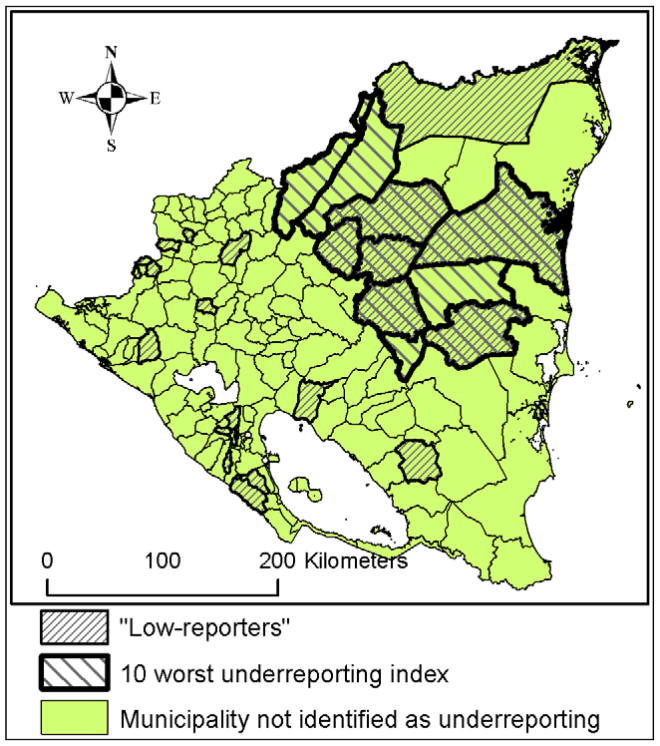
Spatial distribution of municipalities suspected to be underreporting. The 10 municipalities with the worst underreporting indexes and the 24 municipalities identified as “low-reporters” (see Methods section: “Identification of underreporting”).

24 municipalities were identified as “low-reporters”. These were distributed all over the country, whereas the municipalities with the 10 worst scores on the underreporting index were all situated in the north-eastern part of the country (Figure 6). Six out of 24 “low-reporters” were among the 10 municipalities with the worst underreporting index scores.

## Discussion

### Key results

#### Environmental and demographical variables

As expected, different environmental regions have different incidence. The wet and lowland East Coast region has an especially high reported snakebite incidence, probably due to its suitability for the dangerous snake species *Bothrops asper* [10]–[13]. There is a positive association between reported incidence and rural population [3] and to male surplus. Percentage of the population below 15 years of age is negatively associated with snakebite incidence in the adjusted models.

#### Detection of underreporting

As hypothesised, there is an initially positive association between reported incidence and underreporting index followed by a decline in the extreme end, indicating possible underreporting in these areas. Harrison et. al. (2009) [24] described a positive association between poverty and snakebite fatalities in a global analysis at national level. Our findings provide a contrasting perspective: on a sub-national level snakebite incidence might seem negatively associated with poor socioeconomic indicators, potentially because of underreporting. To the best of our knowledge, no study has previously described this relationship or attempted to model underreporting of snakebites.

### Limitations

#### Data gathering

The number of cases *reported* by the health care system might not reflect the true number of cases *treated* by the health care system; individual and/or administrative factors could affect the case reporting rate at the local level (at the specific health care facility). As more “developed” municipalities likely have better reporting routines, this bias may accentuate the negative association between the underreporting index and reported incidence. It was not possible to assess the magnitude of this bias from available data.

When cases reported from hospitals were counted, they were considered coming from the municipality that the hospital is situated in, although these cases could have been referred there from another health care facility in a nearby municipality. This bias would also increase the negative association of the underreporting index and reported incidence since hospitals are situated in municipalities with good underreporting index ranking. 197 (of 3268; 6%) cases were reported from 5 different regional hospitals instead of from municipalities.

The quality of the 2005 census data used in the construction of the underreporting index and the other analyses is unknown, which of course is a major problem. There are data gathering errors during the census to consider, the total population omission was estimated at 3.8–4.5%, but the estimate is uncertain since it was based on a projection from the 1995 census [9]. However, more importantly, the projected population 2007 which was used to calculate the incidence could have be severely affected by rapid and unforeseen migration and thereby constitute a source of error or even bias in this study.

#### Method

The construction of the snake prevalence regions is based on several assumptions and simplifications. Firstly, there probably are municipalities with important differences in snake prevalence within the same region because of differences in altitude and precipitation alone. Secondly, several additional factors, such as the human impact on the environment and the degree of industrialization in agriculture [13] were not included in the construction of the regions because of lack of reliable and available data. The ultimate way to construct the snake prevalence regions would have been to investigate snake prevalence in the each municipality through field observations, but that was clearly outside of the budget of this study. An alternative approach to create the regions with assumed similar snake prevalence could be to use the snake distribution maps available in literature [12]. The regions used in this study correspond relatively well with the snake distribution maps for the two most important snake species.

The extent to which the underreporting index is actually a measure of factors favouring underreporting could be discussed. Human behaviour is immensely complex, but we think that by combining several factors, a context of deprivation and remoteness is described that gives a somewhat more nuanced picture of this complexity than simply focusing on one of the aspects, e.g. poverty.

No information was available on individual factors (age, sex, etc.) of the snakebite victims. Inclusion of variables on this level into the Poisson regression would have enabled a more powerful analysis. With this ecological design, it is not possible to draw conclusions about high risk groups. Our model suggests rural population, men and adults, but these associations could be due to other unmeasured contextual factors associated with the population group and related to incidence and/or reporting rate and thus not necessarily reflect a high incidence within the given population group (a problem known as ecological bias [25]).

### Interpretation

#### Descriptive data

The incidence of 11.2 bites per 100,000 inhabitants/year is similar to the 16 bites per 100,000 inhabitants/year reported from neighbouring Costa Rica [18]. The case fatality rate of 1% is also similar to other countries in the region (e.g. Costa Rica and Panama <1%, Colombia 3–5%) [18]. There are large geographical differences in reported incidence and mortality, as has been noted in studies in neighbouring Costa Rica [26]. Plausible explanations to the seasonal incidence variation are: seasonal differences in snake-human interaction mediated through variations in vegetation density, agricultural activities, and snake behaviour or seasonal variation in case detection and health care accessibility such as variation in road network function (heavy rain can make roads impassable) [27]–[29]. It would be interesting to further investigate the causes of the seasonal variation since this knowledge could provide input to the design of effective preventive strategies, but we consider our present data insufficient for this.

#### Regression model

Although there is a risk of over-interpreting the demographic variables included in this ecological study, we think that rural residency is a risk factor for snakebites in Nicaragua based on our results and strong support in literature [6], [7], [18]. A major part of the association between rural population percentage and reported incidence is removed as the underreporting index is entered as a categorical variable. This shows the strong association between rural residency and poor socio-economic indicators and long distances to healthcare. It also shows that a variable that allows a non-linear relationship between reported incidence and rural conditions absorbs much of the explained variance from a continuous rural population percentage variable. This could be explained if reporting rate decreases with rural population percentage and there thus is a non-linear association between rural population percentage and reported incidence.

Our model indicates that men (or at least inhabitants in municipalities with many men) are more likely to be bitten by snakes, a finding that is supported in literature [17], [18] and a theoretically plausible idea as more men than women work in agriculture [9] where they are likely to encounter a snake. However, municipalities with many men also have a larger rural population, making it very difficult to separate the effects of having a rural versus a male population. When only the percentage of the population under 15 years is included, there is a positive association with incidence, but this changes as other variables are included. There is a positive association between young population and rural population (possibly explaining the positive association to incidence without adjusting for rural population percentage), and also to worse scoring in the underreporting index (possibly explaining the strong negative association before underreporting variable is included). Undoubtedly, studies using individual level data are needed to understand if adult men suffer most from snakebites in Nicaragua or if our findings are merely contextual effects associated with the population group and snakebite incidence and/or reporting rate.

According statistics published by MINSA [17], 31% of all snakebites occur among those under 15 years and 61% among those 15–49 years, an age distribution very similar to that in a review of the epidemiology of *Bothrops asper* bites in Latin America [18]. 38% of the Nicaraguan population is younger than 15 years [9], indicating a somewhat lower snakebite incidence in this age group. However, as noted by the previously mentioned review [18] and a study from neighbouring Costa Rica [30], a significant number of snakebites happen among children, also in Nicaragua [17].

The impact of agricultural and occupational factors on the risk of snakebites was not included in this analysis, although agricultural work is considered a high risk activity for snakebites; Otero-Patiño [18] claims that 85–90% of snakebites are occupational accidents in agricultural fields. Highly industrialized agriculture diminishes snake prevalence [13] and could be assumed to be related to low snakebite incidence. Different types of crops with different cultivating methods and thereby snake-human interaction could affect snakebite incidence. A study using individual-level data is considered necessary for exploring this field.

#### Detection of underreporting

Our interpretation is that the declining incidence ratio in the last underreporting index category is attributable to underreporting; to a certain point case detection is not affected and incidence rises with the index as this is associated with rural population and thereby frequent snake-human encounters and more bites. Eventually, the negative effect on reporting rate cancels out this positive association with high rural population and high *actual* incidence, resulting in a lower *reported* incidence and a drastic dip in the extreme end of the risk-ratio curve for the worst category of the underreporting index.

The two methods used to identify areas where underreporting could be suspected complement each other; method a) identifies areas where there are conditions favouring underreporting and method b) identifies areas where there is a mismatch of exposure, risk group presence and outcome. They also yield different geographical distributions. By design, method b) will identify some municipalities in every region but can never identify if all municipalities in a region could be suspected to be underreporting as this method consists of comparing the municipalities within a region. Method a), on the other hand, can identify entire regions as underreporting as it is not restricted to an intra-regional comparison. It is, however, only sensitive to the most deprived areas.

Relevant questions for this study are the factors that affect snakebite case detection and snakebite victim health care seeking behaviour. The underreporting index that we constructed is a crude attempt to theoretically model this, but more in-depth research is needed to understand this process, establish which variables should be included and how these should be weighted.

Despite the limitations and the lack of verifications by field studies so far, we consider it likely that there are areas where snakebites are underreported in Nicaragua and that our methodology helps identifying these areas. Underreporting of snakebites because of use of traditional medicine is known from similar settings worldwide [4]–[6]. There are reports of traditional treatment of snakebites in Nicaragua [19], [20] as well as personal experiences among the authors of meeting Nicaraguan traditional practitioners claiming to treat snakebites.

We advocate general interventions to increase health care availability and decrease poverty and illiteracy in the areas identified as suspected to be underreporting rather than interventions focusing only on snakebite prevention and treatment.

### Generalizability

A recent study [24] found an association between poverty and snake envenoming on a global level; the poorest countries suffer the most. Our study shows that socioeconomic and health-care related factors are also important to consider when studying snakebite epidemiology on a sub-national level; the relationship between snakebites and e.g. poverty could seem inversed at this level, in our opinion attributable to underreporting.

Secondary data obtained from international organizations such as FAO (Food and Agricultural Organization) have been useful to this study, and the continued availability and improvement of this type of data is important. Geographical Information Systems (GIS) was used extensively as a tool for data preparation and visualization in this study, demonstrating the need for free and efficient GIS-software.

In this study, freely available data about environmental and socioeconomic factors are used to detect areas where underreporting could be suspected. We believe that this creates a less biased map of where further research and interventions addressing snakebites as a health problem are needed. This could be useful in the distribution of resources in the poor settings where snakebites are most common [24] and there is a greater need for a fair interpretation of freely available data.

However, several steps remain before our method can be widely accepted. Field studies in Nicaragua both within the health care system and in communities are necessary to assess the accuracy of our findings and to suggest improvements. Secondly, studies of factors determining snake prevalence and snakebite victim health care seeking behaviour could enable better modelling of these factors, which are fundamental to our analysis but where we were restricted to the use of theoretical assumptions.

## Supporting Information

Table S1 Explanatory variables data sources
(0.04 MB DOC)

Table S2 Altitude, precipitation and snake distribution of environmental regions
(0.04 MB DOC)

Table S3 Underreporting index calculation example
(0.03 MB DOC)

Table S4 Underreporting index categories used in Poisson regression
(0.03 MB DOC)
