# Supplementary material for: Mapping Snakebite Epidemiology in Nicaragua – Pitfalls and Possible Solutions
Source: PLoS Negl Trop Dis. 2010 Nov 23;4(11):e896. doi: 10.1371/journal.pntd.0000896 (PMC2990701; doi:10.1371/journal.pntd.0000896)
Supplement: Table S1 — Explanatory variables data sources (0.04 MB DOC) [file pntd.0000896.s001.doc]

| **Variable (all at municipality level)** | **Data type** | **Source** |
| --- | --- | --- |
| Administrative boundaries | Vector layer | INIDE^2^ |
| **Ecosystem characteristics** | | |
| Topology (average and SD m.a.s.l.) | Raster | FAO^3^ |
| Precipitation (mm/y interpolated to centroid) | Raster | FAO^3,4^ |
| Precipitation (mm/month interpolated to centroid) | Raster | FAO^3^ |
| **Demographic variables** | | |
| Proportion of population rural (%) | Vector layer | INIDE^2^ |
| Male to female ratio (n(male)/n(women) | Vector layer | INIDE^2^ |
| Proportion of population under 15 years old | Vector layer | INIDE^2^ |
| **Socio-economic and health care related indicators** | | |
| Proportion of population in poverty (%) | Table | INIDE^2^ |
| Proportion of population illiterate (%) | Vector layer | INIDE^2^ |
| Number of “birth of last child outside of health care system” per inhabitant^1^ | Table | INIDE^2^ |
| Proportion of households with more than 5 km to health center (%) | Table | INIDE^2^ |
| Proportion of area within 1 km of roads (%) | Vector layer | INIDE^2^ |
| Distance from centroid to hospital (m) | Vector layer | MINSA^5^ |

^1^ Partos del último hijo no atendidos en establecimientos de salud. ^2^ El Instituto Nacional de Información para el Desarrollo (http://www.inide.gob.ni/). ^3^ Food and Agricultural Organization (http://www.fao.org/geonetwork/srv/en/main.home). ^4^ 31 New M, Lister D, Hulme M and Makin I (2000): A high-resolution data set of surface climate over global land areas. Clim. Res., 21:1-25 ^5^Ministerio de Salud, Nicaraguan Ministry of Health (<http://www.minsa.gob.ni/>)
