# Supplementary material for: Mapping Snakebite Epidemiology in Nicaragua – Pitfalls and Possible Solutions
Source: PLoS Negl Trop Dis. 2010 Nov 23;4(11):e896. doi: 10.1371/journal.pntd.0000896 (PMC2990701; doi:10.1371/journal.pntd.0000896)
Supplement: Table S2 — Altitude, precipitation and snake distribution of environmental regions (0.04 MB DOC) [file pntd.0000896.s002.doc]

| **Environmental regions** | **Municipality average altitude (m)** | | | **Precipitation per year (mm)** | | | **No. of municipalities and snake distribution^1^** | | |
| --- | --- | --- | --- | --- | --- | --- | --- | --- | --- |
|  | **Median** | **High** | **Low** | **Median** | **High** | **Low** | **Total** | **% *B.a.*^2^** | **% *C.s.*^3^** |
| East Coast | 83 | 103 | 15 | 3192 | 4244 | 2797 | 18 | 83 | 0 |
| East Inland | 274 | 398 | 74 | 2112 | 2672 | 1753 | 22 | 59 | 14 |
| Mountains | 708 | 1203 | 297 | 1485 | 1822 | 1324 | 56 | 27 | 41 |
| West Coast Central | 177 | 223 | 75 | 1454 | 1526 | 1371 | 19 | 0 | 100 |
| West Coast High | 416 | 603 | 289 | 1486 | 1552 | 1442 | 14 | 0 | 100 |
| West Coast North | 103 | 229 | 10 | 1745 | 1873 | 1574 | 12 | 0 | 100 |
| West Coast South | 106 | 215 | 44 | 1704 | 1803 | 1581 | 11 | 0 | 91 |

1. Percentage of municipalities within environmental region with given snake specie in its center [12] 2. *Bothrops asper* 3. *Crotalus simus*
