# Supplementary material for: Mapping Snakebite Epidemiology in Nicaragua – Pitfalls and Possible Solutions
Source: PLoS Negl Trop Dis. 2010 Nov 23;4(11):e896. doi: 10.1371/journal.pntd.0000896 (PMC2990701; doi:10.1371/journal.pntd.0000896)
Supplement: Table S3 — Underreporting index calculation example (0.03 MB DOC) [file pntd.0000896.s003.doc]

| Munici-pality | Distance to hospital (m) | Households > 5 km from health care center (%) | Population in poverty (%) | Population illiterate (%) | Area of munici-pality >1 km from road (%) | Number of births outside of health care system per inhabitant | Total under-reporting index points |
| --- | --- | --- | --- | --- | --- | --- | --- |
| A | 5 (closest) | 4 | 3 | 5 (least) | 5 (least) | 3 | 25 |
| B | 3 | 5 (least) | 5 (least) | 4 | 3 | 4 | 24 |
| C | 4 | 2 | 4 | 3 | 4 | 5 (least) | 22 |
| D | 2 | 3 | 1 (most) | 2 | 1 (most) | 1 (most) | 10 |
| E | 1 (furthest) | 1 (most) | 2 | 1 (most) | 2 | 2 | 7 |
