# Supplementary material for: Mapping Snakebite Epidemiology in Nicaragua – Pitfalls and Possible Solutions
Source: PLoS Negl Trop Dis. 2010 Nov 23;4(11):e896. doi: 10.1371/journal.pntd.0000896 (PMC2990701; doi:10.1371/journal.pntd.0000896)
Supplement: Table S4 — Underreporting index categories used in Poisson regression (0.03 MB DOC) [file pntd.0000896.s004.doc]

| Category | Number of municipalities | Range of underreporting index | |
| --- | --- | --- | --- |
|  |  | Low | High |
| Worst | 10 | 26 | 107 |
| Bad | 20 | 112 | 245 |
| Medium | 45 | 251 | 457 |
| Good | 45 | 458 | 665 |
| Best | 32 | 666 | 871 |
